# Alleviation of Podophyllotoxin Toxicity Using Coexisting Flavonoids from *Dysosma versipellis*

**DOI:** 10.1371/journal.pone.0072099

**Published:** 2013-08-21

**Authors:** Juan Li, Hua Sun, Lu Jin, Wei Cao, Jin Zhang, Chong-Yi Guo, Ke Ding, Cheng Luo, Wen-Cai Ye, Ren-Wang Jiang

**Affiliations:** 1 Institute of Traditional Chinese Medicine and Natural Products, Jinan University, Guangzhou, China; 2 Key Laboratory of Traditional Chinese Medicine Resource and Compound Prescription, Hubei University of Traditional Chinese Medicine, Wuhan, China; 3 State Key Laboratory of New Drug Research, Shanghai Institute of Materia Medica, Chinese Academy of Sciences, Shanghai, China; 4 Guangzhou Institutes of Biomedicine and Health, Chinese Academy of Sciences, Guangzhou, China; National Cancer Institute at Frederick, United States of America

## Abstract

Podophyllotoxin (POD) is a lignan-type toxin existing in many herbs used in folk medicine. Until now, no effective strategy is available for the management of POD intoxication. This study aims to determine the protective effects of ﬂavonoids (quercetin and kaempferol) on POD-induced toxicity. In Vero cells, both ﬂavonoids protected POD-induced cytotoxicity by recovering alleviating G2/M arrest, decreasing ROS generation and changes of membrane potential, and recovering microtubule structure. In Swiss mice, the group given both POD and ﬂavonoids group had significantly lower mortality rate and showed less damages in the liver and kidney than the group given POD alone. As compared to the POD group, the POD plus ﬂavonoids group exhibited decreases in plasma transaminases, alkaline phosphatase, lactate dehydrogenase, plasma urea, creatinine and malondialdehyde levels, and increases in superoxide dismutase and glutathione levels. Histological examination of the liver and kidney showed less pathological changes in the treatment of POD plus ﬂavonoids group. The protective mechanisms were due to the antioxidant activity of ﬂavonoids against the oxidative stress induced by POD and the competitive binding of ﬂavonoids against POD for the same colchicines-binding sites. The latter binding was confirmed by the tubulin assembly assay in combination with molecular docking analyses. In conclusion, this study for the first time demonstrated that the coexisting flavonoids have great protective effects against the POD toxicity, and results of this study highlighted the great potential of searching for effective antidotes against toxins based on the pharmacological clues.

## Introduction

Although herbal medicines are gaining more acceptances in all over the world, their safety is a major obstacle for further advancement as therapeuticals. Podophyllotoxin (POD), a natural aryltetralin-type lignan, is widely distributed in many plant genera including *Podophyllum* [1], *Dysosma* [2], *Diphylleia* [3] and *Juniperus* [4] of the plant kingdom. These plants have been used in folk medicines because of their potential antitumor [5], antimitotic [6], antiviral [7], and insecticidal [8] activities. However, POD is well known for its potent cytotoxic and neurotoxic properties [9], and is nowadays used as the lead compound for the synthesis of the clinically used anticancer agents etoposide, etopophos and teniposide [10]. POD is a mitotic inhibitor that irreversibly binds to tubulin, and interrupts the dynamic equilibrium between the assembly and disassembly of microtubules, thereby inducing cell cycle arrest at the G2/M phase [5]. Its mode of action is comparable to that of the alkaloid colchicine.

The main deficiencies of podophyllotoxin include its poor selectivity against tumor cells and a narrow therapeutic window. Thus overdose of POD and herbs containing POD often result in serious adverse reactions [11], [12]. In addition, the appearances of the roots of the above plants are similar to Radix Genetianae (gentian roots) and Radix et Rhizoma Clematidis (clematis roots). They can be easily mixed up and further increase the frequency of intoxication incidences of POD [13]. Recently, a series of human poisoning cases have been reported resulting from either overdose or accidental ingestion of podophyllotoxin [14]–[19]. Aside from neurological disturbance [20], patients also exhibited clinical signs and symptoms such as vomiting, diarrhea, abdominal pain and abnormal hepatic functions [14], [21]. Currently, the available therapeutic approaches intend to reduce these symptoms [22] but no specific treatment is available for POD intoxications. Thus discovery of effective antidotes is urgently needed.

Interestingly, not only various podophyllotoxins but also high amount of flavonoids such as quercetin (QUE) and kaempferol (KAE) are coexisting in the same POD containing herbs [1]–[3], [23]. Flavonoids are a group of naturally occurring phenolic compounds widely distributed in the plant kingdom [24]. Similar to *Dysosma versipellis* and *Sinopodophyllum hexandrum* [23], *Catharanthus roseus* also produces high concentration of flavonoids complementary to the toxic vinca alkaloids [25]. In plants, flavonoids may function as detoxifying agents through removing the reactive oxygen species (ROS) and chelating with toxins [26]. Flavonoids are found to locate within or in the proximity of centers of ROS generation in severely stressed plants [27], and the mechanism underlying flavonoid-mediated ROS reduction in plants might be related to the flavonoid-peroxidase reaction [28]. In an ecological context, stress-related oxidative pressure may have been found to be a major trigger for the distribution and abundance of flavonoids [29].

Besides the physiological role in plants, flavonoids also demonstrate displayed interesting pharmacological activities. They have no significant safety concern and exhibit strong antioxidant activity [30]. A large number of epidemiological studies suggest that flavonoids may reduce the incidence of cancers, cardiovascular and all-cause mortality [31]–[33]. Recently the flavonoid QUE was found to bind to tubulin at the same colchicine site as POD [34].

Due to the significant physiological role against the oxidative stress in plants and the pharmacological role of binding to the same colchicine site of tubulin as POD, we hypothesized that the flavonoids coexisting in POD-containing plants might be used to alleviate POD toxicity. In this work we used both *in vitro* and *in vivo* assays to determine whether or not (a) QUE and KAE have any protective effects against POD toxicity, and (b) the protective effects are related to antioxidants.

## Materials and Methods

### Materials

POD, QUE and KAE (purity ≥99%, HPLC grade) were purchased from Aladdin Reagent Co. (Shanghai, China). All cell culture reagents were purchased from Invitrogen (Carlsbad, CA, USA), and cell culture dishes and plates were obtained from Corning Inc. (New York, USA). FITC-conjugated antibody was from Millipore (Billerica, MA, USA). 3-[4,5 dimethylthiazol-2yl]-2,5 diphenyl tetrazolium bromide (MTT), propidium iodide, RNase A, rhodamine 123 (Rh123), 4′,6-diamidino-2-phenyl-indole (DAPI), 2′,7′-dichlorodihydrofluorescein diacetate (DCFH-DA), Rosup and mouse monoclonal anti-tubulin antibody were purchased from Sigma-Aldrich Co. (St. Louis, USA).

### Ethics Statement

This study was conducted in strict accordance with the “Guide for Care and Use of Laboratory Animals” published by the National Institutes of Health and the ethical guidelines of the International Association for the Study of Pain. The protocol was approved by the Animal Care and Use Committee of the Institute of Traditional Chinese Medicine and Natural Products, Jinan University, China (Permit Number: 20100927).

### Cell Culture

Green monkey kidney cells (Vero) were gifts from Prof. Yi-Fei Wang in Jinan University, which was obtained from American type culture collection (Manassas, VA, USA). Cells were maintained as monolayer cultures in RPMI-1640 medium supplemented with 10% fetal bovine serum, 100 units/ml penicillin and streptomycin at 37°C in a 5% CO_2_ humidified atmosphere before tests.

### Cell Viability - MTT Assay

Cell viability was determined using the MTT colorimetric assay [35]. Briefly, 5000 cells per well were seeded into the 96-well plates and allowed to adhere overnight. Cells were then treated with samples at the indicated concentrations. MTT reagent (0.5 mg/ml) was added after 48 h incubation and then incubated for 4 h. Then, culture fluids were removed, 200 µl DMSO was added. Positive drug controls (Paclitaxel) were included to monitor drug sensitivity of each of the cell lines. Results were determined using a microplate reader (TECAN Spectra II Plate Reader, Research Triangle Park, N.C.) at 570 nm.

### Cell Cycle Analysis - Flow Cytometry

To evaluate the percentages of cells at the various stages of the cell cycle, flow cytometric analysis of nuclear DNA content was performed. Following 48 h treatment, cells were harvested with trypsin, washed with PBS, fixed in cold (−20°C) 75% ethanol in PBS, and then stored in 75% ethanol at 4°C. Immediately before FACS analysis, fixed cells were washed with PBS, resuspended in DNA staining solution (0.02 mg/ml propidium iodide and 20 mg/ml RNase A in PBS), and incubated at room temperature for 30 min. Subsequently, cell cycle distribution (DNA histograms) was analyzed by FACSCalibur analysis (Cytomics FC500 ﬂow cytometer, Beckman Colter, Miami, FL, USA). Data analysis was performed using ModFit LT software for cell cycle profiles.

### Analysis of Cell Membrane Potential and Generation of Reactive Oxygen Species (ROS)

The changes of the mitochondrial membrane potential with various treatments in Vero cells were measured by Rh123 using flow cytometer. Briefly, after pretreated with different samples for 48 h, cell membrane potential was measured directly using 10 µg/ml Rh123. Fluorescence was measured after staining the cells for 5–10 min at 37°C. At least 10000 events were evaluated with excitation set at 485 nm and emission monitored at 530 nm.

DCFH-DA is a nonfluorescent compound that is oxidized to the fluorescent 2′,7′-dichlorofluorescin (DCFH) in the presence of oxidants, which could be quantified using a microplate reader. Vero cells were plated into a 24-well plate for 24 h prior the experiment. On the following day, cells were incubated with 10 µM of DCFH-DA at 37°C for 20 min, then cells were rinsed with PBS and were subsequently treated with different samples for about 1 h. As a positive control experiment, cells were treated with 50 mg/ml Rosup for 30 min at 37°C before performing the assay. And the fluorescent signals were measured with 488 nm excitation and 525 nm emission wavelengths using a microplate reader (TECAN Spectra II Plate Reader, Research Triangle Park, N.C.). Experiments were done in triplicate.

### Immunoﬂuorescence Microscopy

Immunoﬂuorescence microscopy was performed as described previously [36]. Brieﬂy, cells seeded on cover-slips in 6-well plates were treated with each compound for 48 h in duplicate. Cells were fixed with 4% paraformaldehyde and nonspecific antibody binding sites were blocked by incubating with 5% BSA in PBS at 37°C for 15 min. Primary antibody for tubulin and fluorescence-labeled secondary antibody were subsequently added to the cells. Cell nuclei were stained by DAPI. Images were taken by using a Confocal Laser Scanning Microscope (Olympus, Japan) with overlays from separate images of tubulin (green) and nuclei (blue).

### Inhibition of Purified Tubulin Assembly

Polymerization assays for purified tubulin were performed using the Tubulin Polymerization Assay kit (BK006P, Cytoskeleton, Denver, CO) according to the instructions of manufacturer. Briefly, tubulin protein (>99% purity, 3 mg/ml) was mixed with different concentrations of POD, QUE, KAE in Tubulin Glycerol Buffer (BST05-001, Cytoskeleton, Denver, CO). Buffer composition was 80 mM piperazine-*N*,*N*′-bis (2-ethanesulfonic acid) sequisodium salt; 2.0 mM magnesium chloride; 0.5 mM ethylene glycol-bis(b-amino-ethyl ether) *N*,*N*,*N*′,*N*′-tetraacetic acid, 60% v/v glycerol, pH 6.9. Microtubule polymerization was monitored at 37°C by light scattering at 340 nm using a multi-well spectrophotometer (Synergy HT, Bio-Tek). The resulting plateau absorbance values were used for all subsequent calculations. 10.0 µM pacilitaxel was used as positive control.

### Molecular Docking

The structures of POD, QUE and KAE were minimized using the Tripos force field and Gasteiger-Hückel charge with distance dependent dielectric and conjugate gradient method with convergence criterion of 0.01 kcal/mol by Sybyl 7.3 (Tripos, Inc). The crystal structure of tubulin protein complex with inhibitor podophyllotoxin was retrieved from Brookhaven Protein Data Bank (PDB entry:1SA1) [37]. Then the GTP molecules, ligand POD and all waters were removed, and the complex composed of protein chains A and B was retained after manual inspection. Hydrogen atoms and charges were added during a brief relaxation performed using the Protein Preparation module in Maestro with the “preparation and refinement” option, and a restrained partial minimization was terminated when the root-mean-square deviation (rmsd) reached a maximum value of 0.3 Å in order to relieve steric-clashes. After the structures of protein, POD, QUE and KAE were carefully prepared, the prediction by Glide was performed with Maestro v7.5 (Schrodinger, Inc.) [38] in extra-precision mode, with up to 10 poses saved per molecule. And the docking results were evaluated by G-score. Finally, the interactions between the POD, QUE and KAE with tubulin protein were carefully analyzed by Pymol and Ligplot (DeLano Scientific, San Carlos, CA, 2002) [39].

### Animals, Experimental Design, Plasma and Tissue Collections

Male Swiss mice (20±2 g) were procured from the Center of Experimental Animals in Sun Yat-sen University, Guangzhou, China. Animals were caged in groups of five and given food and water ad libitum. After one week of acclimation, animals were randomized into 6 groups (10 animals each). The first group was served as the control. Group 2, 3 and 4 were treated with POD (40 mg/kg body weight), QUE (150 mg/kg body weight) and KAE (150 mg/kg body weight), respectively. Group 5 and 6 were treated with POD in combination with QUE or KAE. POD, QUE or KAE were firstly dissolved in dimethyl sulfoxide (DMSO) to prepare the stock solutions and stored at −20°C until use. Then POD, QUE and KAE were diluted with 0.5% carboxymethyl cellulose (CMC) for oral administration. All groups were administered daily for 3 days, and observed for mortality up to 7 days.

Mice were monitored daily for health status, and sacrificed under anesthesia 24 h after the last treatment. This assay was repeated for three times. Blood was drawn from the heart for serum biochemical assays of aspartate aminotransferase (AST), alanine aminotransferase (ALT), lactate dehydrogenase (LDH), blood urea nitrogen (BUN) and creatinine (Cr), and stored at −80°C until assay. Portions of the liver and kidney were immediately removed, weighed and washed using chilled saline solution. For histopathological analysis, one lobe of the liver and right kidney was fixed in 10% formalin. The remaining parts of the liver and kidney were minced and homogenized (10% w/v) in ice-cold 0.15% KCl-0.1 M phosphate buffer (pH 7.4) in a Potter-Elvehjem homogenizer. The tissue homogenates were stored at −20°C for the assays of alkaline phosphatase (AKP), level of reduced glutathione (GSH), malondialdehyde (MDA), superoxide dismutase (SOD) and protein content.

### Histopathological Section Preparation

For each mouse, three specimens from different regions of liver and kidney were collected and fixed in 10% formalin in PBS at room temperature overnight. Dehydration of tissue specimens in ascending grades of alcohol was performed. Impregnation of tissues in melted paraffin wax was done in the oven at 60°C for one hour. Tissues were embedded with melted hard paraffin, then left to solidify at room temperature to form blocks. The paraffin-embedded tissue sections (5 mm) were stained with hematoxylin and eosin (H&E) using standard techniques. Examination of sections from all groups under light microscope and assessment of various groups was performed.

### Biochemical Parameters

The activities of AST, ALT, LDH, Cr and BUN in serum and the levels of AKP, MDA, SOD and GSH in liver homogenate were determined photometrically in accordance with the manufacturer’s protocol by using commercially available enzymatic assay kits (Nanjing Jiancheng Bioengineering Institute, Nanjing City, P. R. China). All the experiments described in this section were performed in triplicate to obtain means and standard deviations (SD).

### Statistical Evaluation

Data were expressed as mean ± SD. Statistical analysis was carried out using SPSS 18.0 software. Statistical significance was determined by Student’s *t* test. A probability of less than or equal to 0.05 was considered to be statistically significant.

## Results

### Effect of QUE, KAE, POD and their Co-treatment on Cell Viability Changes

**Figure 1:**
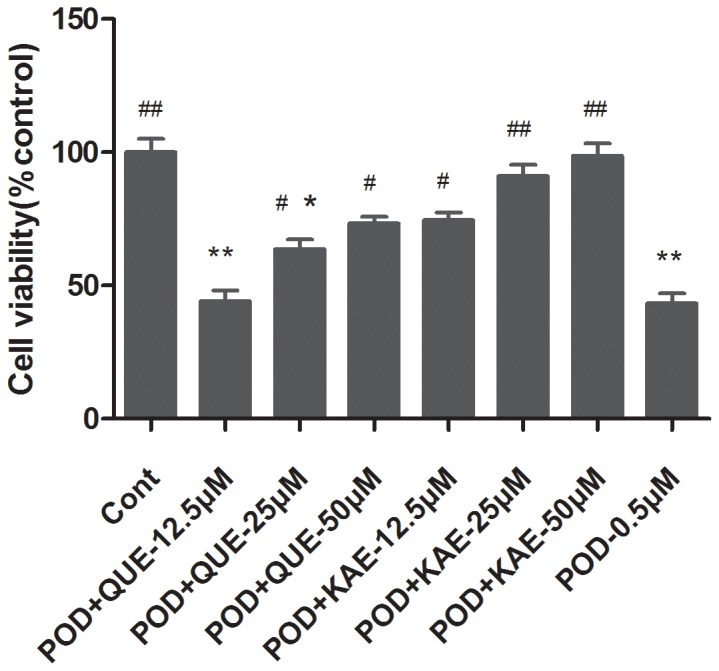
Effect of QUE, KAE, POD and their co-treatment on cell viability changes in Vero cells in 48 h. Cell viability was determined by MTT assay. The cell viability of cells treated with 0.5 µM POD was reduced compared to the control. A significant increase of cell viability was observed when cells were co-treated with QUE or KAE. Cont-control, POD-podophyllotoxin, QUE-quercetin, KAE-kaempferol, *p≤0.05 compared to the control group, **p≤0.01 compared to the control group, **^#^**p≤0.05 compared to the POD group, **^##^**p≤0.01 compared to the POD group.

The protective effects of QUE and KAE against POD-induced cell death were measured in cultured Vero cells by MTT. Cells were either treated with POD alone or co-treated with QUE and KAE for 48 h. POD alone significantly inhibited the cell viability with an IC_50_ of ∼0.47 µM. At 0.5 µM, POD showed ∼43.3% cell viability. Therefore, 0.5 µM POD was chosen for subsequent experiments. However, the viability of cells showed no significant change when they were treated with QUE or KAE up to 50 µM as compared to that of the control. As shown in Fig. 1, a significant increase of cell viability was observed when cells were co-treated with POD and QUE (>25 µM) or POD and KAE (>12.5 µM) as compared with POD alone, and this protective effect was in a dose independent manner. Taken together, it suggests that POD induced a significant inhibition of proliferation in Vero cells, while QUE and KAE had a protective effect on POD-induced cytotoxicity.

### Protective Effects of QUE and KAE on POD Induced Cell Cycle Arrest

**Figure 2:**
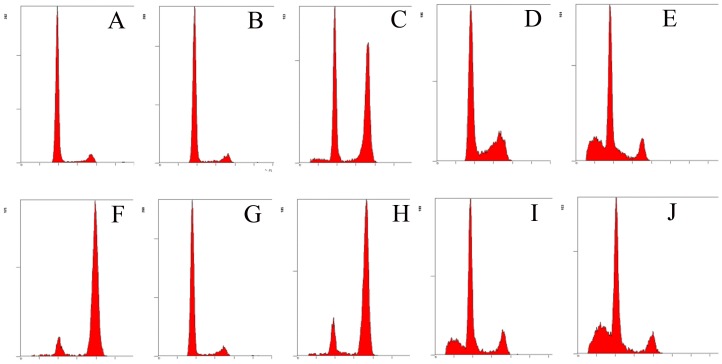
Protective effects of QUE and KAE on POD induced cell cycle arrest. A: control group, B: KAE-50 µM, C: POD-0.5 µM +KAE-12.5 µM, D: POD-0.5 µM +KAE-25 µM, E: POD-0.5 µM +KAE-50 µM, F: POD-0.5 µM, G: QUE-50 µM, H: POD-0.5 µM +QUE-12.5 µM, I: POD-0.5 µM +QUE-25 µM, J: POD-0.5 µM + QUE-50 µM. After 48 h exposure, cells were harvested and stained with PI, and the cell cycle distribution was analyzed by flow cytometry. POD-podophyllotoxin, QUE-quercetin, KAE-kaempferol.

Uncontrolled cell division can lead to programmed cell death. The percentages of cell cycle were determined by flow cytometry. After a 48 h treatment exposure to 0.5 µM POD, ∼66.6% of cells were observed at G2/M phase as compared with ∼10.2% of control cells (Fig. 2). Thus POD caused a significant G2/M phase arrest. In contrast, the cell cycle distributions of either QUE- or KAE-treated cells seemed similar to the vehicle controls. In addition, the ratios of cells at G2/M phase were reduced to ∼46.0%, 12.0%, 9.3% by POD co-treatment with QUE in the concentration of 12.5 µM, 25 µM and 50 µM, and to ∼43.4%, 13.2%, 8.7% by POD co-treatment with KAE at the concentrations of 12.5 µM, 25 µM and 50 µM, respectively (Fig. 2). Thus, both QUE and KAE had protective effects on POD-induced cell cycle arrest at G2/M phase.

### Protective Effects of QUE and KAE on POD-induced Changes of Cell Membrane Potential

**Figure 3:**
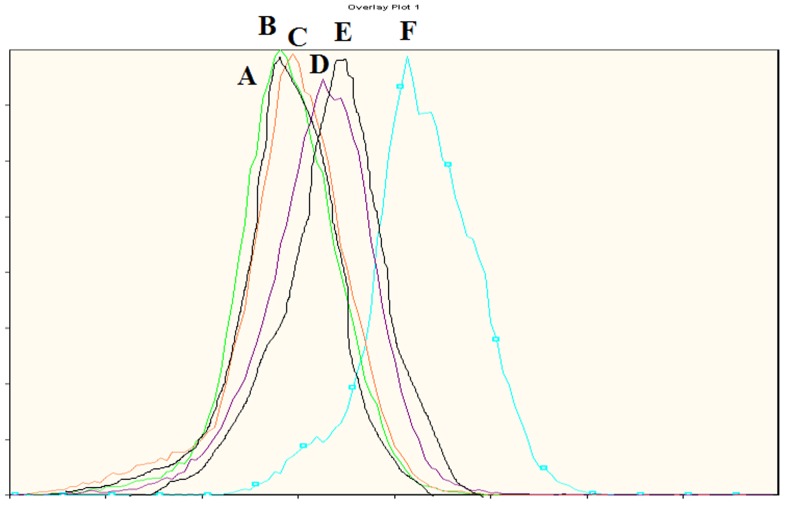
Protective effects of QUE and KAE on POD induced change of cell membrane potential. A: control group. B: QUE-20 µM. C: KAE-20 µM. D: POD-0.5 µM + QUE-20 µM; E: POD-0.5 µM +KAE-20 µM; F: POD-0.5 µM. Cells were harvested after treated 48 h and stained with Rh123, and the cell membrane potential was analyzed by flow cytometry.

Mitochondrial membrane potential is a marker of mitochondria functions, and is often associated with ROS generation. The cell membrane potential was measured based on the mean fluorescence intensity of live Vero cells through Rh123 staining. As shown in Fig. 3 higher level of fluorescence was achieved after a 48 h exposure to 0.5 µM of POD (∼36.8) as compared with the control (∼9.5). Meanwhile, the fluorescence level of either QUE- and KAE-treated cells seemed similar to that of the vehicle controls. The fluorescence levels for POD (0.5 µM) co-treatment with QUE (20 µM) or KAE (20 µM) were ∼21.1 and ∼21.4, respectively (Fig. 3). In concordance with MTT result and cell cycle analyses shown above, co-treatment of QUE or KAE could decrease POD-induced increase of fluorescence level in a dose dependent manner. Therefore, QUE and KAE had protective effects on POD-induced changes of cell membrane potential.

### QUE and KAE Suppressed POD-induced ROS Generation

**Figure 4:**
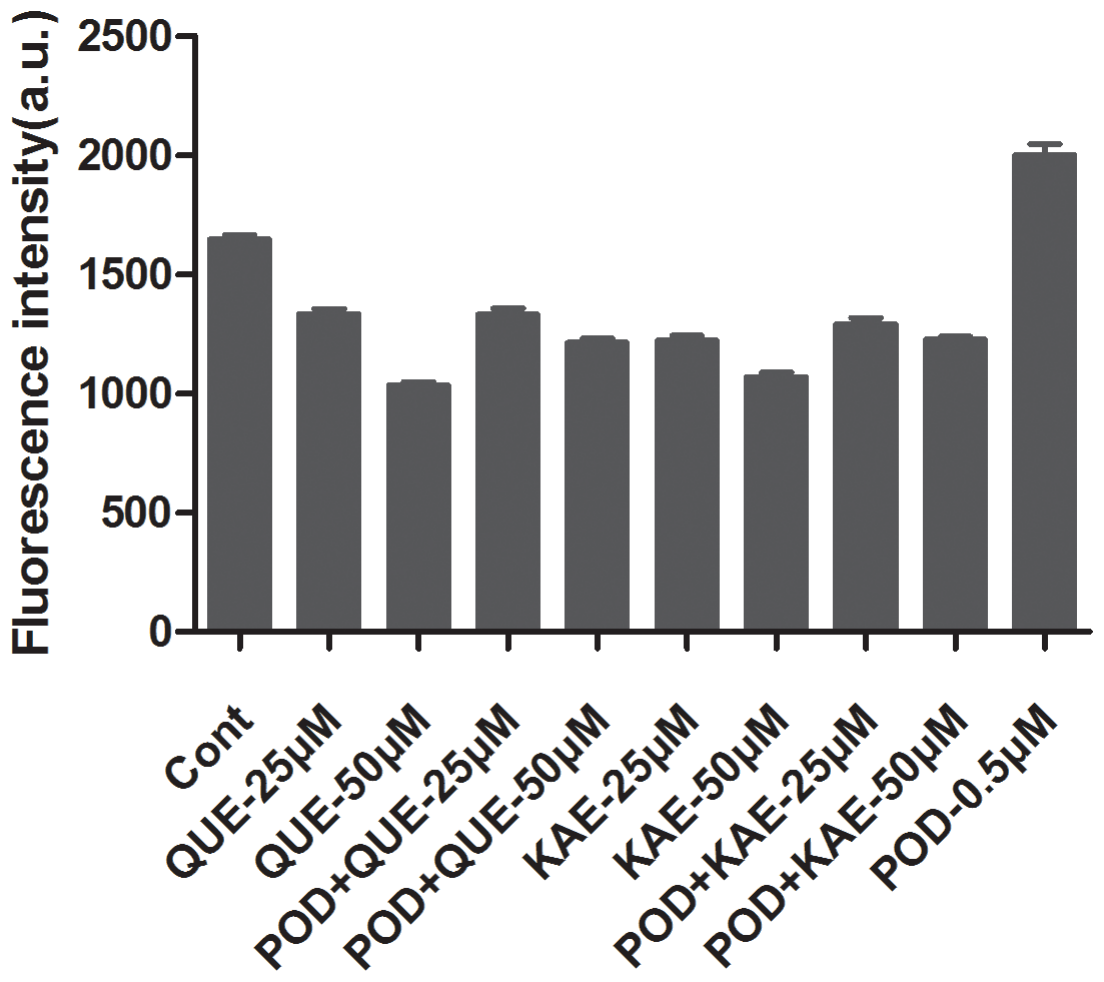
QUE and KAE suppressed POD induced ROS generation. Quantifications of ROS in Vero cells determined by the oxidation of DCFH-DA dye. Cont-control, POD-podophyllotoxin, QUE-quercetin, KAE-kaempferol.

Since ROS played an important role in cell death and the change of mitochondrial membrane potential was considered to be related to ROS production, we next investigated the intracellular ROS formation using a fluorescent sensitive probe (DCFH-DA). As presented in Fig. 4, the generation of ROS was due to POD toxicity. POD at 0.5 µM increased ROS level by about 1.5-fold when compared with the control. Simultaneous treatment with QUE or KAE with POD caused a decrease in the ROS level as compared to the POD alone, and sometimes even reduced ROS below the basal level, which was similar to the treatment with either QUE or KAE alone. Therefore, POD-induced ROS generation could be blocked by flavonoids at both 25 and 50 µM.

### Effects of QUE and KAE on POD-induced Microtubules Depolymerization

**Figure 5:**
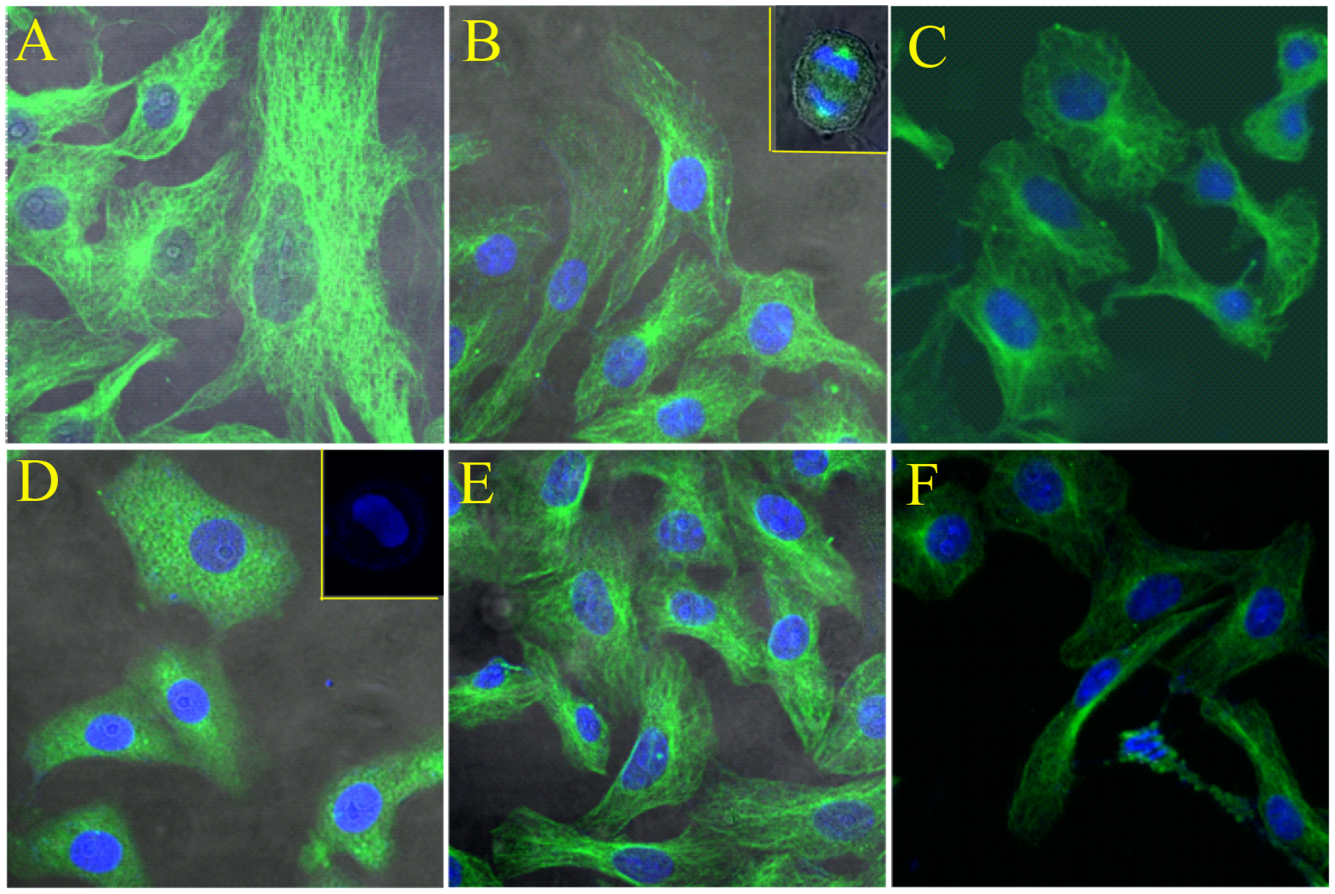
The effects of QUE and KAE on the interphase microtubules of Vero cells examined using immunoﬂuorescence microscopy. A: Control group; B: QUE-50 µM; C: POD-0.5 µM +QUE-50 µM; D: POD-0.5 µM; E: KAE-50 µM; F: POD-0.5 µM +KAE-50 µM. POD-podophyllotoxin, QUE-quercetin, KAE-kaempferol.

The effects of QUE and KAE on the interphase microtubules of Vero cells were examined using immunoﬂuorescence microscopy (Fig. 5). Control cells showed typical interphase microtubule organization. As shown in Fig. 5, treatment of cells with 0.5 µM POD for 48 h led to a complete depolymerization of the microtubule cytoskeleton. Some cells were micronucleated or arrested in prometaphase with a ball or rosette of condensed DNA and no mitotic spindle was observed [6]. The POD-induced mitotic arrest was accompanied by net microtubule depolymerization. However, the effect of 50 µM either QUE or KAE on the interphase microtubule network was not apparent. Meanwhile, the QUE or KAE in combination with POD group had a significant higher microtubule density than the POD alone group. Therefore, QUE and KAE could antagonize the POD-induced microtubules depolymerization.

**Figure 6:**
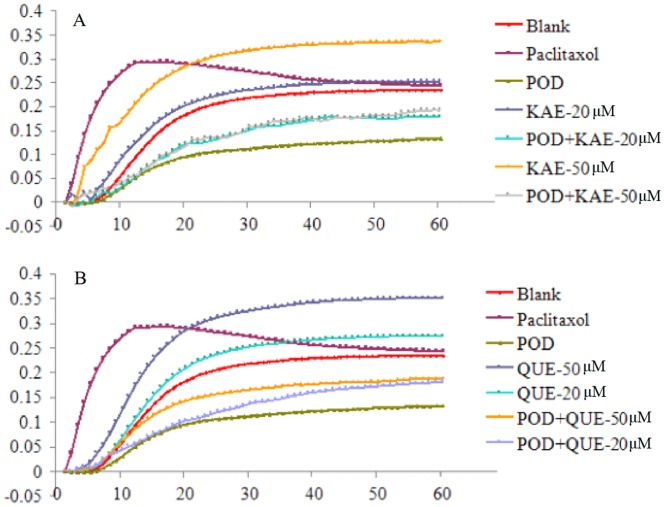
Effect of various concentration of QUE, KAE, POD and their co-treatment on microtubule polymerization. Paclitaxel was used as positive control. POD-podophyllotoxin, QUE-quercetin, KAE-kaempferol.

Polymerization assays on purified tubulin were performed by a tubulin polymerization assay kit. POD showed inhibitory effect on tubulin polymerization in a dose-dependent manner. As shown in Fig. 6, POD exhibited about 50% inhibitory activities on tubulin polymerization at 5.0 µM. Interestingly, QUE or KAE enhanced the tubulin polymerization at concentrations of 20.0 µM and 50.0 µM. The lines of combination groups, POD (5.0 µM)+QUE (20.0 µM), POD (5.0 µM)+KAE (20.0 µM), POD (5.0 µM)+QUE(50.0 µM), and POD (5.0 µM)+KAE (50.0 µM), were between the lines of POD, QUE and KAE, and the inhibition rates were about 22.2%, 23.5%, 17.9%, 19.7%, respectively. These results implied that QUE and KAE could decrease POD-induced inhibition of tubulin polymerization.

### Comparison of the Interaction Modes between the POD, QUE or KAE with Tubulin

**Figure 7:**
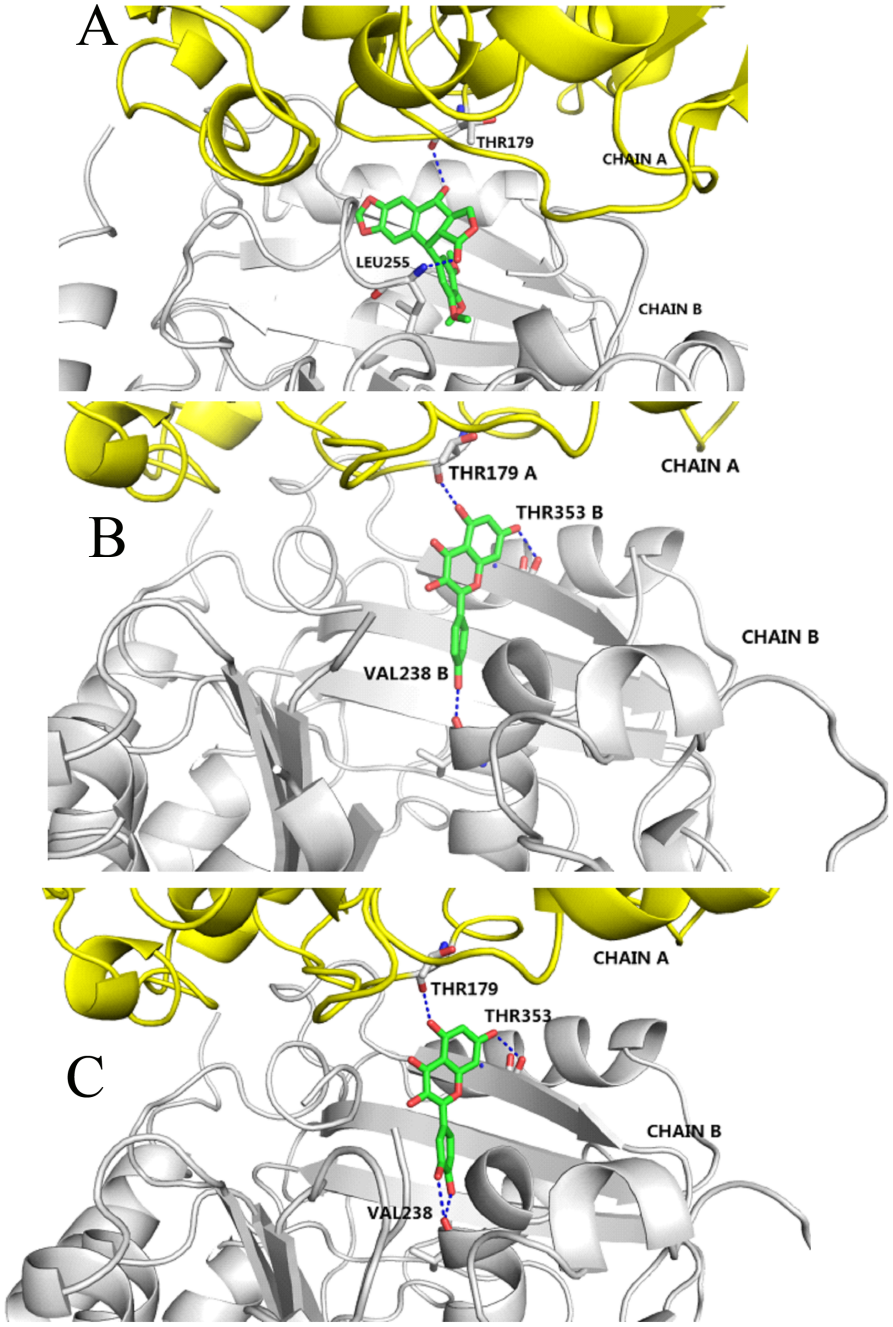
The binding mode of POD, KAE or QUE with tubulin. (A) POD. (B) KAE. (C) QUE. All interactions were analyzed by Pymol and Ligplot. POD-podophyllotoxin, QUE-quercetin, KAE-kaempferol.

We compared the binding modes of tubulin with POD, QUE or KAE. The modeling data of POD (Fig. 7A) indicated that the C-4 hydroxyl group hbonded with the carboxyl group of Thr179 in chain A and C-12 carbonyl group hbonded with the backbone of Leu255 in chain B. While in the tubulin/QUE complex (Fig. 7C), the 5-OH and 7-OH groups hbonded with Thr179 in chain A and Thr353 in chain B, respectively, and both 3′-OH and 4′-OH in the phenyl ring hbonded with Val238 in chain B. Similarly, in the tubulin/KAE complex (Fig. 7B), 5-OH and 7-OH groups also hbonded with Thr179 in chain A and Thr353 in chain B, respectively, and the only 4′-OH hbonded with Val238 in chain B. The docking G-score for POD-, QUE- and KAE-docking results of G-score were −9.78, −9.08, and −8.16, respectively. Thus, though POD, QUE and KAE exhibited strong interactions with tubulin, they adopted different binding modes, which would account for the antagonizing effect of QUE or KAE against the POD-induced microtubules depolymerization.

### Effects of QUE, KAE, POD and their Co-treatment on Mortality, Food Intake, and Body Weights of Mice

The POD-treated (40 mg/kg) mice started to die on Day 3 after treatment, and the total mortality was 80.0% on Day 7. In the co-treatment groups (40 mg/kg POD plus 150 mg/kg QUE or KAE), first animal death was observed on Day 4 and the total mortality was 20% and 30% mortality on Day 7, respectively. No death was observed in mice treated with QUE or KAE (150 mg/kg) alone.

**Figure 8:**
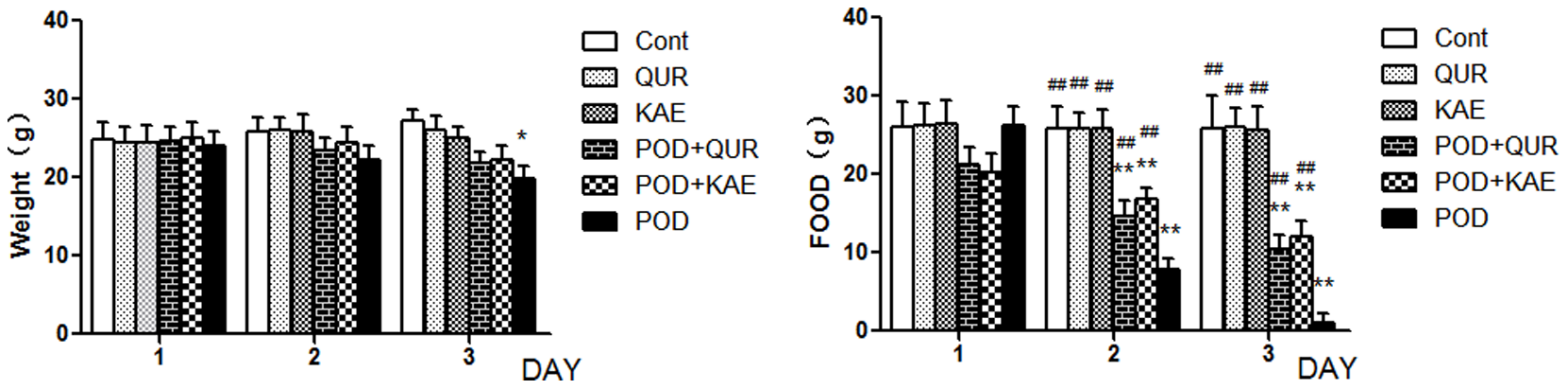
Effects of QUE, KAE, POD and their co-treatment on food taken, body weights of mice. Cont-control, POD-podophyllotoxin, QUE-quercetin, KAE-kaempferol. *p≤0.05 compared to the control group, **p≤0.01 compared to the control group, **^#^**p≤0.05 compared to the POD group, **^##^**p≤0.01 compared to the POD group.

Body weights and food intake were monitored throughout the experiment as indications of adverse effects. Exposure to POD in mice caused significantly decrease in body weight (27.0%) and food intaken (95.9%) as compared with the control group (Fig. 8). No significant effects on body weight and food intake were observed in mice treated with QUE or KAE as compared to the control. The decreases of body weights (19.5%, 18.4%) and food intakes (59.3%, 53.1%) in the groups given POD plus QUE or KAE were much less than those of the POD group; these results indicated less toxicity of the co-treatment groups (Fig. 8). Thus, QUE and KAE could improve the health status in POD-poisoning.

### Histological Examination

**Figure 9:**
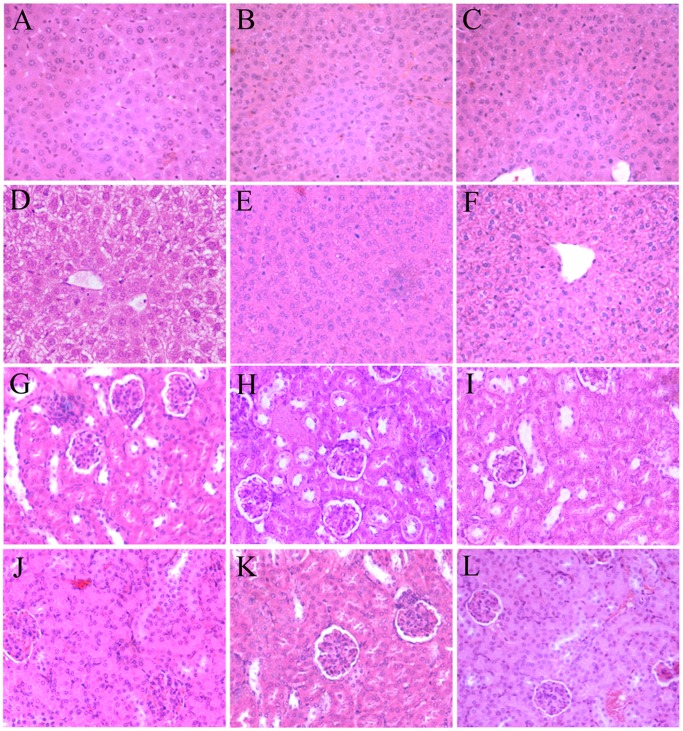
Histological changes of the liver and kidney tissues treated with POD alone and in combination with flavonoids. (A) Control of liver tissues. (B) QUE treatment of liver tissues. (C) KAE treatment of liver tissues. (D) POD treatment of liver tissues. (E) POD+QUE treatment of liver tissues. (F) POD+KAE treatment of liver tissues. (G) Control of kindney tissues. (H) QUE treatment of kindney tissues. (I) KAE treatment of kindney tissues. (J) POD treatment of kindney tissues. (K) POD+QUE treatment of kindney tissues. (L) POD+KAE treatment of kindney tissues. Sections were stained with hematoxylin and eosin (400×). POD-podophyllotoxin, QUE-quercetin, KAE-kaempferol.

Examination of liver sections of the untreated group revealed normal hepatic architecture with cords of hepatocytes radiating from the central vein. The hepatocytes appeared polyhedral in shape with well defined boundaries and granular cytoplasm. Each cell exhibited a round vesicular, centrally located nucleus and open sinusoidal spaces (Fig. 9A). Groups treated with QUE or KAE alone showed the same normal histology of control (Fig. 9B, C). Liver sections of mice that received POD alone revealed vacuolated with fatty degeneration, particularly the cells of the periportal regions (Fig. 9D). Administration of QUE in combination with POD resulted in satisfactory protection against POD-induced fatty degeneration. The classical hepatic architecture was preserved with apparently normal hepatocytes (Fig. 9E). Meanwhile, concomitant administration of KAE with POD resulted in partial preservation of general histological appearance of liver. Many hepatocytes still exhibited vacuolated cytoplasm (Fig. 9F).

Similarly, the transverse sections of the kidneys in the control group showed well-developed glomerulus with normal tubular cells (Fig. 9G). Groups treated with QUE or KAE alone showed the same normal histology as in the control (Fig. 9H, I). Kidney sections of mice that received POD alone showed swelling of glomeruli, decrease in the Bowman capsule, and increase in tubular cells (Fig. 9J). Co-administration of QUE with POD resulted in apparently preservation of general histological appearance of kidney with better developed glomerulus than the POD POD-treated group (Fig. 9K). While, administration of KAE with POD resulted in partial preservation against POD-induced glomerulus changes (Fig. 9L).

### Protective Effects on Liver Functions

**Figure 10:**
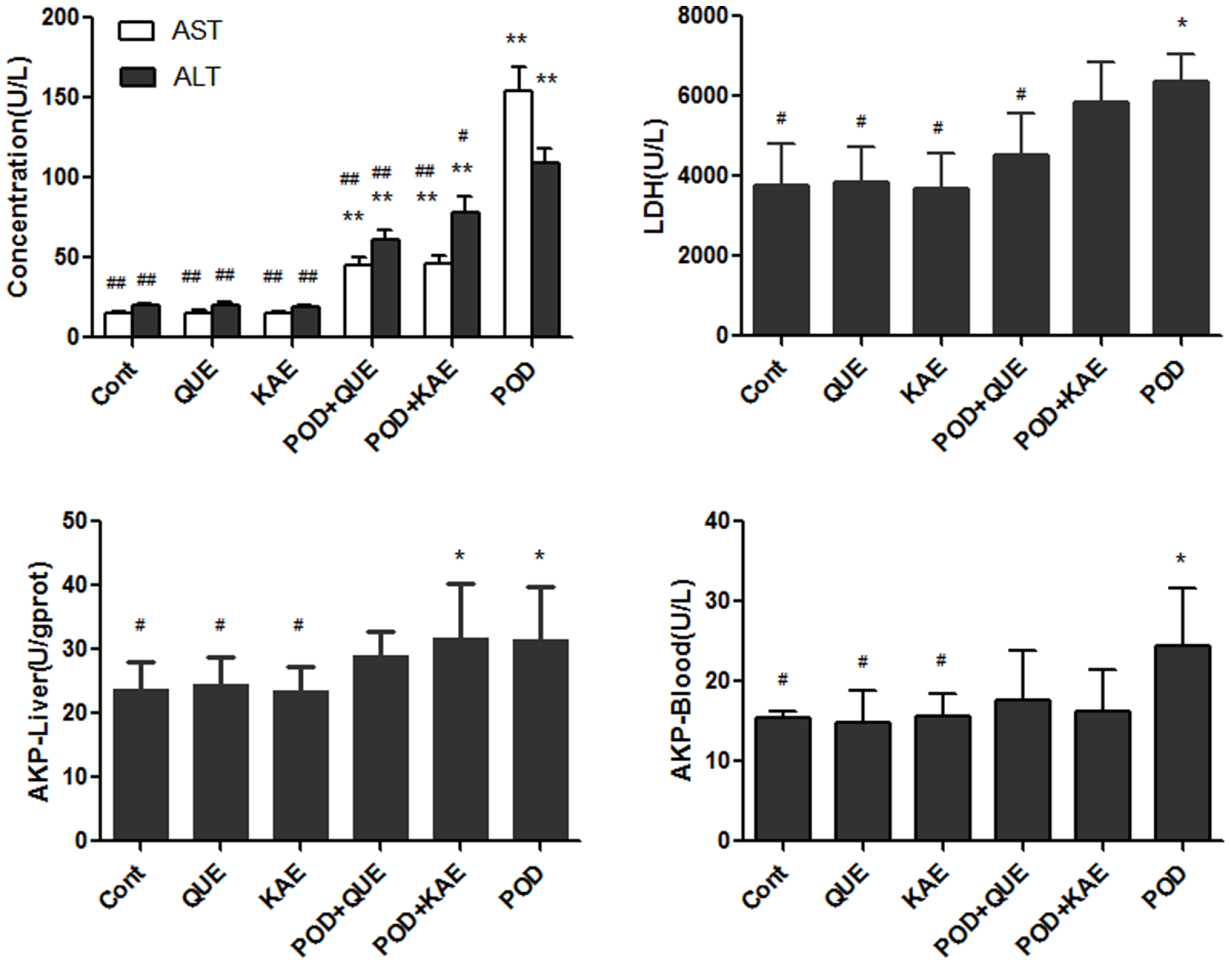
Protective effects of QUE and KAE on POD induced liver damages. Cont-control, POD-podophyllotoxin, QUE-quercetin, KAE-kaempferol, AKP-alkaline phosphatase, ALT-alanine aminotransferase, AST-aspartate aminotransferase, LDH-lactate dehydrogenase, *p≤0.05 compared to the control group, **p≤0.01 compared to the control group, **^#^**p≤0.05 compared to the POD group, **^##^**p≤0.01 compared to the POD group.

Hepatotoxicity is generally associated with elevated plasma levels of ALT, AST, LDH and AKP [40], [41]. Treatment with POD for 3 days resulted in a significant (p<0.05) increase in the levels of ALT (5.5 fold), AST (10.2 fold), LDH (1.7 fold), AKP in serum (1.6 fold) and AKP in liver tissue (1.3 fold) as compared to the control. Interestingly, simultaneous treatment with QUE and POD in mice caused decreases in the activities of ALT (55.6%), AST (29.3%), LDH (71.1%) and AKP in serum (71.7%) as compared to mice treated with POD alone. Similarly, simultaneous treatment with KAE and POD in mice caused decreases in the activities of ALT (71.6%), AST (30.2%), LDH (92.1%) and AKP in serum (66.6%) as compared to mice treated with POD alone. No significant changes in the ALT, AST, LDH and AKP activities were observed in mice treated with QUE or KAE as compared to controls. (Fig. 10, Table 1).

**Table 1:** Protective effects of QUE and KAE on Liver function in mice^a^.

Group	AST(U/L)	ALT(U/L)	AKP–Serum(U/L)	AKP-Liver(U/gprot)	LDH (U/L)
Control	14.97±0.99##	19.94±0.84##	15.52±0.80#	23.83±4.24#	3778.02±1037.92#
QUE	15.64±1.56##	20.32±1.47##	14.89±3.86#	24.47±4.22#	3829.04±902.50#
KAE	15.07±1.23##	19.58±0.97##	15.73±2.79#	23.64±3.53#	3689.06±873.76#
POD+QUE	45.12±4.97** ##	60.74±6.17** ##	17.55±6.35	28.93±3.81	4521.74±1032.67#
POD+KAE	46.56±4.36** ##	78.22±9.47** #	16.29±5.06	31.87±8.51*	5854.32±991.02
POD	154.01±14.75**	109.17±8.85**	24.47±7.07*	31.44±8.23*	6357.84±674.30*

a Mice were given a dose of QUE (150 mg/kg), KAE (150 mg/kg), POD (40 mg/kg), POD (40 mg/kg)+QUE(150 mg/kg), POD (40 mg/kg)+KAE (150 mg/kg), treated by intragastrical administration for 3 days. Mice were sacrificed under anesthesia 24 h after the last treatment. Values represent mean±SE.

* indicates significantly different from control at p≤0.05.

** indicates significantly different from control at p≤0.01.

# indicates significantly different from POD group at p≤0.05.

## indicates significantly different from POD group at p≤0.01.

### Protective Effects on Kidney Functions

**Figure 11:**
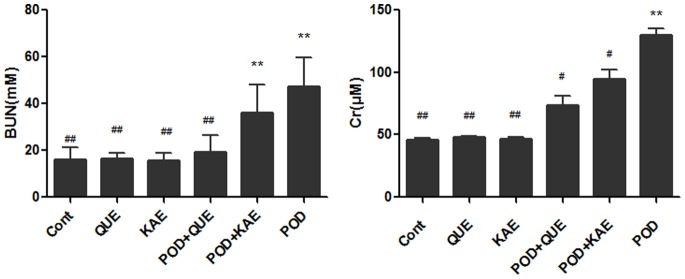
Protective effects of QUE and KAE on POD induced kidney damages. Cont-control, POD-podophyllotoxin, QUE-quercetin, KAE-kaempferol, BUN-blood urea nitrogen, Cr-creatinine, *p≤0.05 compared to the control group, **p≤0.01 compared to the control group, **^#^**p≤0.05 compared to the POD group, **^##^** p≤0.01 compared to the POD group.

Kidney functions were monitored by measuring the plasma levels of BUN and Cr. The results indicated that POD caused a significant (p<0.05) increases in BUN and Cr levels. Treatment with QUE or KAE alone did not affect the tested parameters. However, in the combinations with POD, QUE significantly alleviated the distortion in these biochemical parameters, suggesting an amelioration of the impairment of kidney functions. However, KAE resulted in no significant protection against POD-induced kidney functional changes (Fig. 11, Table 2).

**Table 2:** Protective effects of QUE and KAE on Kidney function in mice^a^.

Group	BUN(mM)	Cr( µM)
Control	15.92±5.37##	45.87±1.52##
QUE	16.34±2.39##	47.78±0.93##
KAE	15.59±3.23##	46.72±1.03##
POD+QUE	19.08±7.19##	73.66±7.70#
POD+KAE	36.07±11.80**	94.77±7.62#
POD	47.17±12.42**	130.19±5.09**

a Mice were given a dose of QUE (150 mg/kg), KAE (150 mg/kg), POD (40 mg/kg), POD (40 mg/kg)+QUE(150 mg/kg), POD (40 mg/kg)+KAE (150 mg/kg), treated by intragastrical administration for 3 days. Mice were sacrificed under anesthesia 24 h after the last treatment. Values represent mean ± SE.

* indicates significantly different from control at p≤0.05.

** indicates significantly different from control at p≤0.01.

# indicates significantly different from POD group at p≤0.05.

## indicates significantly different from POD group at p≤0.01.

### Effects on Oxidative Stress

**Figure 12:**
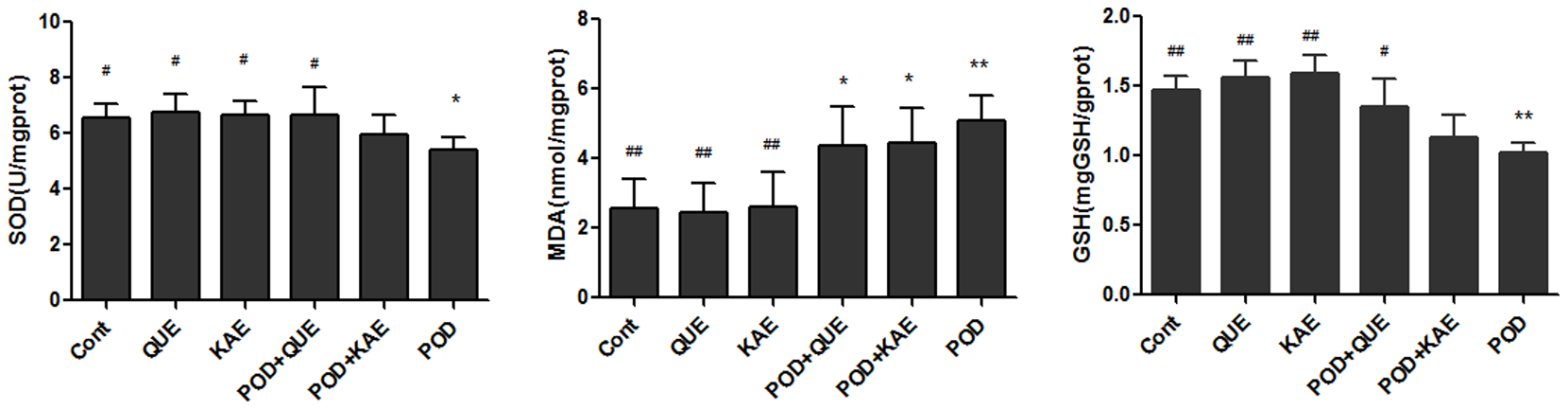
Protective effects of QUE and KAE on POD induced oxidative stress. Cont-control, POD-podophyllotoxin, QUE-quercetin, KAE- kaempferol, GSH-glutathione, MDA-malondialdehyde, SOD-superoxide dismutase, *p≤0.05 compared to the control group, **p≤0.01 compared to the control group, **^#^**p≤0.05 compared to the POD group, **^##^** p≤0.01 compared to the POD group.

Exposure to POD significantly decreased the SOD and GSH levels but increased the MDA level in the liver tissues as compared with the control group (Fig. 12 and Table 3). Treatment with QUE or KAE alone did not affect any of these tested parameters. However, simultaneous treatment QUE with POD in mice caused a decrease in the activity of MDA level but increased SOD and GSH levels as compared with the POD group. But, KAE had no significant protection against the oxidative injury (Fig. 12, Table 3).

**Table 3:** Effects of QUE and KAE treatment on POD induced oxidative stress in mice^a^.

Group	MDA (nmol/mgprot)	SOD (U/mgprot)	GSH (mg GSH/gprot)
Control	2.58±0.81##	6.55±0.52#	1.47±0.10##
QUE	2.46±0.82##	6.76±0.64#	1.56±0.12##
KAE	2.62±0.97##	6.64±0.53#	1.59±0.13##
POD+QUE	4.36±1.11*	6.68±0.97#	1.35±0.20#
POD+KAE	4.44±1.02*	5.98±0.67	1.13±0.16
POD	5.08±0.71**	5.42±0.45*	1.02±0.07**

a Mice were given a dose of QUE (150 mg/kg), KAE (150 mg/kg), POD (40 mg/kg), POD (40 mg/kg)+QUE(150 mg/kg), POD (40 mg/kg)+KAE (150 mg/kg), treated by intragastrical administration for 3 days. Mice were sacrificed under anesthesia 24 h after the last treatment. Values represent mean±SE.

* indicates significantly different from control at p≤0.05.

** indicates significantly different from control at p≤0.01.

# indicates significantly different from POD group at p≤0.05.

## indicates significantly different from POD group at p≤0.01.

## Discussion

It has been estimated by the World Health Organization that approximately 80% of the world’s population were using folk medicine for primary health care [42], and these folk medicines were often administrated without prescriptions. Plants containing POD and its analogues were used in folk medicines as agents for treating snake bites [21], [43], cancer, viral infection and astriction [44]. Because of its poor selectivity and small therapeutic index, POD has been incriminated in many poisoning cases as a result of either overdose or accidental ingestion [14]–[16], [18], [19]. Major disturbances include symptoms in gastrointestinal tract such as vomiting, diarrhea and abdominal pain [14], [21] and sometimes even neurological disorders [44]. Oxidative damages and inhibition of mitosis (disruption of microtubules) are believed to be the underlying mechanisms of these changes observed in the animals intoxicated by POD [45]. The available therapeutic approaches include induction of vomiting, gastric lavage, infusion, fluid and electrolyte replacement, cardiovascular and respiratory support [21]. So far, no specific antidote is available for treating POD intoxications.

During our on-going project to search for effective antidotes from natural products, we had reported on the promising role of curcumin as a protective agent against the POD poisoning [46]. Considering the physiological (co-existence of flavonoids and POD in *Dysosma versipellis* [2], the antioxidant properties of flavonoids in plants) and the pharmacological roles (tubulin-binding properties), the protective effects of QUE and KAE were investigated. We found that co-treatment with QUE or KAE significant decreased POD induced cell death, ROS generation and changes of membrane potential, and antagonized the POD-induced inhibition of the tubulin polymerization, suggesting that QUE and KAE could offer protections by preserving the structural integrity of the Vero cells and competing for the POD-binding sites on tubulin, which was further confirmed by the subsequent molecular docking study. The docking G-score of POD, QUE and KAE were −9.78, −9.08 and −8.16, respectively, suggesting that both QUE and KAE had lower but comparable binding affinity to tubulin as POD. Furthermore, high doses of QUE or KAE might induce a conformational change of tubulin, which might decrease the binding affinity of POD. It is noteworthy that both KAE and QUE enhanced tubulin-polymerization but showed no effect on the cell cycle in normal Vero cells used in this study. The normal cells are different from the cancer cells. It was reported that exposure to 50 µM KAE in some cancer cells (HL-60, PC3, human hepatic cancer cells) [47]–[49] could increase the proportion of cells in the G2-M phase and a decrease in the proportions of cells in the G1 and S-phases. In contrast phytoestrogens including QUE were found to be safe for the Vero cells up to 50 µM [50].

Significant increases in mortality, histopathological changes and related biochemical markers, such as ALT, AST, AKP, LDH, BUN and Cr, were observed after the POD treatment, indicating the presence of serious hepatotoxicity [40] and renal toxicity [51]. Co-treatment with POD with either QUE or KAE significantly decreased the mortality rate of Swiss mice and the corresponding biochemical markers in the liver and kidney. Thus our results showed that QUE and KAE could provide both *in vitro* and *in vivo* protections against POD toxicity. In addition, treatment of POD also led to serious neurological disorders as revealed by the unsteady movement behavior. Co-treatment with QUE or KAE significantly improved the movement behavior (data not shown), which was consistent with the prevous report that KUE (20 and 40 mg/kg, p.o.) had a neuroprotective effect against colchicine-induced cognitive dysfunctions and oxidative damage [52].

The protective properties of flavonoids against toxins have also been reported in numerous experimental studies, particularly against oxidative damages induced by OP pesticides [53]–[55]. QUE can attenuate liver injury [56] and renal impairment [57], [58], and provide neuroprotective protection against excitotoxic insults [59], [60]. KAE can also provide cardio- and neuro-protections [61]. Though QUE or KAE have been reported to exhibit protective effects against a variety of toxins through different mechanisms, the results here show for the first time that they offered protections against POD intoxication through interactions with the tubulin.

Similar to carbon tetrachloride [62], POD intoxication involve significant oxidative stress. Flavonoids like QUE and KAE are well known antioxidants through proton-donation from the phenolic hydroxyl groups [63]. In addition, flavonoids could target on the oxidative signaling pathways and directly interact with the endogenous antioxidant defense system [64]. For example, flavonoids could inhibit several phase I enzymes (mainly the CYP450 complex enzymes), and could induce phase II detoxifying enzymes, such as quinone reductase, glutathione transferases, glucuronyl transferases and xanthine oxidase [63], [65]. In the present study, co-treatment of POD with either QUE or KAE significantly decreased the ROS generation and MDA levels but increased the SOD and GSH levels, indicating a significantly improved oxidative status. In the present study, QUE exhibited a more potent protection than KAE and the previously reported curcumin [46], which might be due to its stronger antioxidant activity [28].

Our study proved that flavonoids have protective effects on POD toxicity; however, the detailed signaling pathway remains unclear. It appears that the activation of Cdk1 through Cdc25C, together with up-regulation of cyclinB1, contributes to POD-induced G2/M cell cycle arrest; the activation of ATM, Chk2 and p53 might contribute to POD-induced apoptosis [66]. Continued presence of flavonoids in the cellular environment may attenuate the activity of tubulin-acting agents. The interference of QUE (12.5–50 µM) can diminish the efficacy of tubulin-targeting drugs (taxol and nocodazole) to arrest cells at G2/M, due to the combination treatment that prevents accumulation of Cyclin-B1 at the microtubule organizing center (MTOC) [67]. Flavonoids can also delay microtubules depolymerization and induce the formation of small aggregates [68]. In addition, flavonoids could increase the microtubule stability through interactions with microtubule-associated proteins (MAP) such as tau, MAP1B, and MAP2c [69]–[71].

Bioavailability is worthy of note when considering flavonoids as the potential antidotes. Recent studies have shown that a considerable amount of dietary QUE can be absorbed through the digestive tract [72] and undergo subsequent metabolic conversion in hepatic and intestinal tissues [73]. Autoradiographic analyses of rats three hours after receiving QUE-treatment showed that most of the radioactivity remained in the digestive tract [74]. The plasma levels in rats with chronic dietary intake of QUE (50 mg/kg body weight) exceeded 20 µM [75]. Similar to QUE, KAE is also mainly absorbed in the small intestine [61]. Thus the distribution profiles of QUE and KAE are consistent with that of POD, which is also mainly distributed in the gastrointestinal tract. Thus the local bioavailability of QUE and KAE (p.o.) in the organs might be sufficient to provide effective concentration rescuing the toxicities of POD, which were confirmed in our studies. It should be noted that considering the low oral bioavailability of flavonoids, a high concentration of flavonoids (150 mg/kg) was used in this study, and our results prove that flavonoids have protective effects on POD toxicity. It can be stressed that improving metabolic stability of these compounds is feasible (through new delivery system, for example) and could be a useful approach for improving oral bioavailability and therefore the dosages of flavonoids could be decreased [76].

Currently, a number of plant-derived natural toxins and their derivatives were used in clinics as anticancer drugs [77]. Though these drugs showed benefits for the cancer treatment, the therapeutic index was narrow and the treatment process was often accompanied by severe toxic side effects [78]. Knowledges of the toxic pattern and mechanism and available antidotes were required before application of these drugs. However, for most of these anticancer drugs, effective antidotes or specific strategies were still not available except for some symptomatic treatment [79]. The results of this study showed that it was an useful approach to search for effective antidotes against the toxins based on the ecological and pharmacoloigcal clues from natural products.

## Conclusions

The present study clearly demonstrated that a) QUE or KAE protected against POD-induced cytotoxicity by recovering G2/M arrest and reducing the change of membrane potential, assembly of tubulin, and ROS generation; b) QUE or KAE could decrease the mortality rate of rats and protect the liver and kidney functions with decreased ALT, AST, AKP, LDH, Cr, BUN and MDA levels, but elevated SOD and GSH activity, and QUE was found to exhibit a more pronounced protection *in vivo* than KAE; and c) the protective effects might be due to the competitive binding of QUE or KAE with POD in the same colchicines binding site and the antioxidant activities against the oxidative stress induced by POD. In summary, QUE or KAE had protective effects against POD toxicity both *in vitro* and *in vivo*. They could be used as potential antidotes to treat the POD intoxication in clinical practice. Results of this study highlighted the great potential of searching for effective antidotes against toxins based on the plant physiological and pharmacological information of toxic herbs.
